# Synthesis and Characterization of a Nanoscale Hyaluronic Acid-Specific Probe for Magnetic Particle Imaging and Magnetic Resonance Imaging

**DOI:** 10.3390/nano15191505

**Published:** 2025-10-01

**Authors:** Harald Kratz, Dietmar Eberbeck, Frank Wiekhorst, Matthias Taupitz, Jörg Schnorr

**Affiliations:** 1 Department of Radiology, Charité-Universitätsmedizin Berlin, Corporate Member of Freie Universität Berlin and Humboldt-Universität zu Berlin, D-10117 Berlin, Germany; matthias.taupitz@charite.de (M.T.); joerg.schnorr@charite.de (J.S.); 2 Physikalisch-Technische Bundesanstalt, D-10587 Berlin, Germany; dietmar.eberbeck@ptb.de (D.E.); frank.wiekhorst@ptb.de (F.W.)

**Keywords:** extracellular matrix, glycosaminoglycans, hyaluronic acid, chondroitin sulfate, iron oxide nanoparticles, quartz crystal microbalance

## Abstract

Glycosaminoglycans (GAGs) are part of the extracellular matrix (ECM) and play a major role in maintaining their physiological function. During pathological processes, the ECM is remodeled and its GAG composition changes. Hyaluronic acid (HA) is one of the GAGs that plays an important role in pathological processes such as inflammation and cancer and is therefore an interesting target for imaging. To provide iron oxide nanoparticles (IONP) that bind to hyaluronic acid (HA) as specific probes for molecular imaging, a peptide with high affinity for HA was covalently bound to the surface of commercial IONP (synomag^®^-D, NH2) leading to hyaluronic acid-specific iron oxide nanoparticles (HAIONPs). Affinity measurements using a quartz crystal microbalance (QCM) showed a very high affinity of HAIONP to HA, but not to the control chondroitin sulfate (CS). HAIONPs exhibit a very high magnetic particle spectroscopy (MPS) signal amplitude, which predestines them as HA-selective tracers for magnetic particle imaging (MPI). The high relaxivity coefficient *r*_2_ also makes HAIONP suitable for magnetic resonance imaging (MRI) applications. HAIONP therefore offers excellent prerequisites for further development as a probe for the specific quantitative imaging of the HA content of the ECM in pathological areas.

## 1. Introduction

Glycosaminoglycans (GAGs) are one of the major parts of the extracellular matrix (ECM); they show a high water-binding capacity and have a variety of functions in the body, e.g., cell signaling, lubrication and shock absorption [1], tissue repair and homeostasis. GAGs also play a major role in pathological processes like tumor progression, angiogenesis and inflammation [2,3,4,5]. They are polymers consisting of different disaccharide units of N-acetylglucosamine, N-acetylgalactosamine or galactose, with glucuronic acid or iduronic acid. The six most important types of GAGs in the body are chondroitin sulfate (CS), heparin, heparan sulfate, dermatan sulfate, keratan sulfate and hyaluronic acid (HA) [6]. HA is the only GAG that is not covalently conjugated to proteins and is not sulfated. All other GAGs are part of proteoglycans, which means that they are covalently bound to proteins [6]. In addition, they are sulfated at one or more hydroxyl groups, resulting in different sulfation patterns [5,7]. A 70 kg person has approx. 15 g of hyaluronan in the body, of which about 5 g is metabolized daily [8]. HA is synthesized on the plasma membrane by a family of three HA synthases (HAS1, HAS2 and HAS3) [9]. After synthesis, it is released to the extracellular space or remains in the pericellular space bound to HA-binding proteins or HAS. HA can interact with several cell surface proteins which act as HA receptors like CD44, RHAMM [9], ICAM-1 [10] and LYVE-1 [9]. Under physiological conditions, HA has a high average molecular weight of over 10^6^ Da, is anti-inflammatory and supports normal homeostasis. HA with a low molecular weight acts in a pro-inflammatory and pro-angiogenic way and therefore is associated with inflammation, tumorigenesis or tissue injury, and its appearance is a signal that the normal homeostasis has been profoundly disturbed [4,11,12]. In terms of synthesis, HAS2 is one of the main factors for the increased extracellular deposition of low molecular weight HA in a variety of inflammatory diseases, creating a microenvironment that favors angiogenesis and inflammation and promotes disease progression. In breast cancer, overexpression of HAS2 is involved in tumor growth and differentiation, and metastatic spread to the axillary lymph nodes [12]. Due to the diverse functions of HA in the body, many of which are not yet fully understood, it is important to develop new imaging tools to investigate the remodeling processes of the ECM and, in particular, the function of HA in various diseases in more detail. It seems difficult to generate HA-specific antibodies [13], but several peptides and proteins with a high affinity for HA have been described in the literature [13,14,15,16,17]. This generally opens up the possibility of developing new HA-specific imaging probes, e.g., as a tracer for magnetic particle imaging (MPI) [18,19] or as a contrast agent for magnetic resonance imaging (MRI) [20,21]. The aim of this project was to develop such HA-specific probes on the base of iron oxide nanoparticles (IONPs) with the perspective to detect pathological ECM changes. In the following sections, we describe the synthesis of HA-specific iron oxide nanoparticles (HAIONP) by coupling HA-specific peptides (HASP) on the surface of a commercial nanoparticle system (synomag-D, NH2) for potential use in MPI and MRI [14,22,23,24,25], and also their physicochemical characterization as well as HA affinity studies.

## 2. Materials and Methods

### 2.1. Chemicals

Unless otherwise noted, all chemicals were purchased from Sigma-Aldrich (Steinheim, Germany) in high purity (≥98%) and were used without any further purification. Deionized water was produced using a Milli-Q A10 system (Millipore, Billerica, MA, USA) and was used to prepare all solutions and dispersions. Phosphate-buffered saline (PBS) buffer and PBS-ethylenediaminetetraacetic acid (EDTA) buffer 1× and 10× (pH 7.45) were prepared using gibco PBS Tablets (ThermoFischer Scientific, Waltham, MA, USA) Na_2_EDTA and the corresponding amounts of water (500 mL for 1× and 50 mL for 10×). Sulfosuccinimidyl-*trans*-4-(N-maleimidomethyl)cyclohexane-1-carboxylate (Sulfo-SMCC) was purchased from AmBeed, Arlington, TX, USA). Hyaluronic acid 50K (HA) and chondroitin sulfate 50K (CS) were purchased from Creative PEG Works, NC, USA. N-Hydroxysuccinimide (≥99%), L-cysteine (≥99%) and 5,5′-Dithio-bis-(2-nitrobenzoic acid) (≥98%) were purchased from Carl Roth GmbH (Karlsruhe, Germany). HASP, modified IP3-peptide (CGGKRDLSRRA, purity ≥ 95%), was synthesized by Charité Peptide Synthesis Facility via solid phase synthesis. For dialysis, SpectraPor^®^ 4 tubes with a cutoff of 12–14 kDa (Repligen, Waltham, MA, USA) were used after pre-wetting with water for 15 min.

### 2.2. IONP

Commercially available IONPs (synomag^®^-D; surface: NH2; diameter 50 nm, order number: 104-01-501, micromod Partikeltechnologie GmbH, Rostock, Germany) were used for surface modification with HASP. The IONPs consist of several smaller maghemite crystals in one nanoparticle (magnetic multi-cores) coated by a dextran matrix with added amino groups (-NH_2_) on the surface and, according to the manufacturer, have a mean hydrodynamic diameter of *d*_hyd_ = 50 nm by intensity. All experiments were performed with the same pooled synomag^®^-D dispersion.

### 2.3. Purification of IONP Intermediates and HAIONPs by Magnetic Separation During Synthesis

For purification of IONP intermediates and HAIONPs after the individual synthesis steps, LS Columns (Miltenyi Biotec, Bergisch Gladbach, Germany) were used combined with a MidiMACS^®^ Separator (Miltenyi Biotec, Bergisch Gladbach, Germany). The LS Columns were each equilibrated twice with 5 mL of the corresponding buffer before use. The magnetic purification/separation procedure was carried out using Technote-101_4 (Technote-101_4.pdf), which can be found on the micromod.de website and was adapted accordingly.

### 2.4. Analytic Measurements

Analytical iron and thiol determinations were carried out using a UV-VIS spectrophotometer (Genesys 6 spectrophotometer, Thermo Fisher Scientific, Waltham, MA, USA). The thiol content of glycosaminoglycan-cysteine conjugates was determined photometrically using an Ellman assay [26,27] with L-cysteine as standard and measured at 412 nm. The measurement was carried out as a triple determination and the evaluation was based on a calibration curve. The iron concentration c(Fe) of IONP was determined spectrophotometrically by dissolution with 6N HCl and hydrogen peroxide for 30 min at room temperature (RT) and subsequent measurement of the Fe(III) complex formed at 410 nm. The measurement was carried out as a triple determination and the evaluation was based on a calibration curve [28]. FTIR measurements were performed using a Bruker ALPHA spectrometer (model A250/D, Bruker Optik GmbH, Leipzig, Germany) equipped with a diamond ATR sampling module (model A220/D-01, Bruker Optik GmbH, Leipzig, Germany). Measured data were processed using the OPUS 6.5 software package (Bruker Optik GmbH, Leipzig, Germany). Background and baseline correction as well as atmospheric compensation were applied to all spectra. Spectra were acquired in absorbance mode and converted to transmission mode from 23 coadded scans between 4000 and 375 cm^−1^.

### 2.5. Nuclear Magnetic Resonance (NMR)-Relaxivities

^1^H-NMR *T*_1_- and *T*_2_-relaxation rates of synomag^®^-D and HAIONP were measured with a Minispec MQ 60 time-domain NMR (TD-NMR) spectrometer (Bruker, Karlsruhe, Germany) at *T* = 37 °C and *B*_0_ = 1.41 T. Relaxivities (relaxation coefficients) *r*_1_ and *r*_2_ were determined by the linear fitting of *T*_1_- and *T*_2_-relaxation rates as functions of iron concentrations *c*(Fe). *T*_1_ was measured using a spin inversion recovery sequence and *T*_2_ using a Carr_Purcelll- Meiboom-Gill (CPMG) sequence; both were evaluated by monoexponential curve fitting.

### 2.6. Magnetic Particle Spectroscopy (MPS) and Direct Current Magnetization (DCM) Characterization

IONPs were also analyzed by magnetic particle spectroscopy (MPS) to assess the nonlinear dynamic response of the magnetic moments of IONPs to an alternating magnetic field *B*_0_. MPS measurements were performed using a magnetic particle spectrometer (MPS-3, Bruker BioSpin, Ettlingen, Germany) at *B*_0_ = 25 mT, *f*_0_ = 25 kHz, *V* = 30 μL, and *T* = 37 °C. The amplitude of the magnetic moment was represented as a spectrum of amplitudes of multiples *k* of the base frequency *f*_0_, *M*_k_. *M*_k_ was normalized to the iron content of the sample yielding the unit Am^2^/mol(Fe). For DC magnetization measurements (*M*(*H*) measurements), *V* = 50 μL sample volumes were filled into polycarbonate capsules. The induced magnetic moment of the sample was measured using a SQUID magnetometer (Magnetic Property Measurement System MPMS-XL, Quantum Design, San Diego, CA, USA) successively increasing the applied magnetic field *H* from 0 to 5 T. The background signal caused by empty polycarbonate capsules and the diamagnetic susceptibility of the dispersion medium was subtracted from the sample signal. The resulting magnetic moment was translated into the magnetization *M* dividing the moment by sample volume. For the sake of quantitative comparison, *M* was normalized to the volume fraction of the magnetic component *ϕ*.

### 2.7. Dynamic Light Scattering (DLS) and ζ-Potential

Hydrodynamic diameters and ζ-potentials of IONPs were determined by DLS on a Zetasizer Nano ZS particle analyzer (Malvern Instruments, Worcestershire, UK) and measured at 23 °C (size) and 25 °C (ζ-potential). For DLS measurement, IONP dispersions were diluted with Milli-Q water to a final concentration of c(Fe) = 1 mmol Fe/L. For ζ-potential measurement, IONP dispersions were diluted with 10 mM NaCl to a final concentration of c(Fe) = 1 mmol Fe/L and adjusted to a pH value of 7.40 with NaOH and HCl. Six measurements were performed for the size measurements and three for the zeta potential.

### 2.8. Bicinchoninic Acid (BCA) Assay to Determine the Amount of HA Specific Peptide Incorporated Within HAIONPs

For peptide determination of HAIONPs, a Pierce BCA protein assay kit was used (Thermo Fisher Scientific, Waltham, MA, USA). For the calibration curve, the synomag^®^-D parent IONPs were added to increasing HASP concentrations at the same iron concentration as in the measured HAIONPs. The measurement was carried out as a triple determination and the evaluation was performed using a calibration curve.

### 2.9. Quartz Crystal Microbalance (QCM) Device

QCM measurements were implemented using a QCM (QCM NEXT QCM Device (Novaetech S.r.l, Pompei, Italy) using gold plated 10 MHz Quartz sensors for liquid biosensing (Novaetech S.r.l, Pompei, Italy) and controlled by openQCM software (version 0.1.5 for WIN OS 64 bit). The sensors have a blank diameter of 14 mm, a gold electrode diameter of 11.5 mm and a total thickness of about 160 µm. In addition to the fundamental frequency, two overtones were measured simultaneously (3rd and 5th). For the flow experiments a clinical infusion syringe pump (BD Alaris GH Plus, BD Medical, Franklin Lakes, NJ, USA) with single-use syringes from 5 to 20 mL (BD Discardit II, Becton Dickinson, Franklin Lakes, NJ, USA) connected with Tygon tubes (1/16″ ID Tygon^®^ 2375 tubing, AJK00002 from Saint-Gobain, Courbevoie, France) via tube connectors (Fisherbrand^®^ Female Luer with 1/16 in. ID Barb-Polypropylene-QC from Fisher Scientific GmbH, Schwerte, Germany) were used. Piranha solution for sensor cleaning was prepared by carefully mixing 10 mL of concentrated sulfuric acid with 3 mL of 30% hydrogen peroxide. The sensor was treated with this freshly prepared piranha solution for just 30 s to avoid oxidation of the gold surface and then rinsed three times with water, three times with absolute ethanol and dried with compressed air [29]. For QCM measurement PBS was degassed for 2 min under a pressure of 30 kPa. All solutions and dispersions were prepared using this degassed PBS.

### 2.10. QCM Measurement and Evaluation

We designed our system for measuring the binding affinity so that two different glycosaminoglycans, HA for affinity tests and CS as a control, were bound to the sensor surface. Thiol groups form strong bonds to gold surfaces, the bond strength of which can be up to 40–50 kcal/mol depending on the given pH and other factors, i.e., in the range of covalent bonds [30]. Due to the increase in mass during the binding process, a decrease in the sensor oscillation frequency can be observed in the QCM. In addition to the frequency shift, the dissipation of the applied vibrational energy is also an important parameter, which is a measure of the viscoelastic properties of the layer of deposited molecules [31]. For determination of dissipation, the sensor is set into oscillation followed by measurement of the decay of the oscillations after removal of excitation, i.e., how it is damped [32]. When thin, rigid layers are deposited, the dissipation changes little or not at all. With viscoelastic layers, a clear change in dissipation can be observed [33]. Due to the high specific mass of HAIONPs, the frequency decrease in case of a binding to HA on the surface should be relatively high. All QCM experiments were performed at a flow rate of 5 mL/h using a clinical infusion syringe pump and repeated at least three times. First, the system was run with PBS until the baseline stabilized. This is normally the case after about an hour. Then the sensor was coated with solutions of the respective thiolated glycosaminoglycans HA-cys or CS-cys at a concentration of 1 mg/mL in PBS for 12 min. The potentially free binding sites on the gold surface were then blocked with L-cysteine to prevent non-specific binding of the HAIONPs. This was performed with a L-cysteine solution (1 mg/mL) in PBS for 24 min. After that the sensor was treated with HAIONPs in PBS (0.0333 mg Fe/mL) for 100 min and the frequency decrease was measured. The coated sensor was then rinsed with PBS for 25 min to check the stability of the binding of the HAIONP to the HA or CS bound to the gold surface. Between individual coating steps, the sensor was washed with PBS for 24 min to clean the surface of unbound substances and to avoid direct interactions between the different coating components. In addition, a baseline was measured to accurately determine the frequency change Δ*f* by the following coating. We used the third overtone for evaluation since it exhibited the lowest proportion of interference.

### 2.11. Preparation of Samples for MPS Adsorption Measurements

#### 2.11.1. Adsorption of HAIONPs on GAG-Coated QCM Sensors, Static Without Flow

To roughly estimate the strength and specificity of the HAIONP’s HA binding, we used MPS to analyze the concentration changes in HAIONP dispersion droplets pipetted onto HA-cys and CS-cys (control) coated QCM sensor surfaces, respectively. For this purpose, two gold plated QCM sensors were treated either with (a). 1 mL HA-cys PBS solution (1 mg/mL) or with (b). CS-cys PBS solution (1 mg/mL) at RT for 195 min under an argon atmosphere on a neoLabLine Rocking Shaker (neoLab Migge GmbH, Heidelberg, Germany). Then, both differently coated sensors were treated with 2 mL L-cysteine PBS solution (1 mg/mL) for 80 min at RT under an argon atmosphere and then washed three times each with PBS, water and absolute ethanol and then dried under a stream of air. Afterwards, 30 µL of a HAIONP stock dispersion (HAIONP in PBS (*c*(Fe) = 0.6 mmol/L)) were pipetted onto the middle of the gold surface of the two GAG-coated sensors. After 60 min at RT, the supernatant was pipetted off again in both cases (dispersions: HA and CS). A volume of 30 µL PBS was then pipetted onto the center of each GAG-coated sensor and then immediately pipetted off (dispersions: HA-wash and CS-wash). The five dispersions (HAIONP stock, HA, CS, HA-wash and CS-wash) were subsequently measured by MPS to determine and compare the IONP content in the different dispersions.

#### 2.11.2. Adsorption of HAIONPs on GAG-Coated QCM Sensors After QCM Affinity Measurements

In order to confirm the different amounts of HAIONPs adsorbed on the QCM sensors using another method, we carefully removed the HA-cys and CS-cys coated QCM sensors after the measurements and then removed the HAIONP from the surface with a piece of cellulose tissue, then measured it with MPS. For this purpose, precision wipes (Kimtech Science^®^, Kimberly-Clark professional^®^, Koblenz, Germany) were cut into 15 × 10 mm pieces using a ceramic scalpel.

### 2.12. MPS Measurement of the Adsorption of HAIONPs on GAG-Coated QCM Sensors Without Flow and QCM Sensors After Affinity Measurement

Magnetic particle spectroscopy (MPS) is based on detecting the nonlinear dynamic response of the magnetic moments of the MNP when exposed to an oscillating magnetic excitation field [34]. Taking only the nonlinear components of the magnetization response into consideration enables the detection and quantification of specific IONPs only. The technique is extremely sensitive, capable of detecting dynamic magnetic moments down to 5 pAm^2^. The response of the MNP is recorded in the time-domain and then processed using Fourier transformation and averaging, yielding the characteristic MPS amplitude *A*_n_ and phase *ϕ*_n_ spectra, where the dominant response occurs at odd harmonics (i.e., n = 3, 5, 7, etc.) of the excitation frequency *f*_ex_. The harmonic amplitudes *A*_n_ of the MPS spectrum are directly proportional to the amount of IONPs (or amount of IONP iron), with the third harmonic amplitude *A*_3_ typically used for quantification. First, the MPS spectrum of reference samples of known iron amount *m*(Fe) are measured from which the specific moment *A*_3_* (moment normalized to the iron amount in units Am^2^/kg (Fe)) can be determined for each IONP system. Depending on the requirements for the samples to be measured, the reference samples may consist of IONPs in aqueous dispersion, or the IONPs may be immobilized using plaster (Moltofill Spachtelpulver innen, Akzo Nobel Deco GmbH, Köln, Germany). Then, the iron amount of the sample to be quantified is obtained by dividing the measured *A*_3_ moment by *A*_3_*. MPS measurements of dispersions and cellulose tissues of the HAIONP adsorption experiments were performed using a magnetic particle spectrometer (MPS-3, Bruker BioSpin, Ettlingen, Germany) at *B*_0_ = 25 mT, *f*_ex_ = 25 kHz, *V* = 10 μL, and *T* = 37 °C.

### 2.13. Preparation of IONP Control Sample (IONPC) by Treatment of Synomag^®^-D with Magnetic LS Columns

For the preparation of the IONPC, 2.5 mL of synomag^®^-D (*c*(Fe) = 92.6 mmol/L) were mixed with 275 µL of 10× PBS-EDTA buffer (100 mM sodium phosphates, 10 mM Na_2_EDTA) and 170 µL DMSO and stirred for 60 min at RT. Thereafter the dispersion was filled into an LS Column which is placed in a MidiMACS^®^ Separator and pre-washed twice with 5 mL PBS-EDTA-buffer 1×. Then the IONP-loaded LS Column was washed with 5 mL PBS-EDTA-buffer 1×, removed from the MidiMACS^®^ Separator and converted synomag^®^-D were eluted with 5 mL PBS-EDTA-buffer 1×. A 2.60 mL main fraction was obtained. Pre-run and post-run were each discarded. The main fraction was then mixed with 1.5 mL PBS-EDTA-buffer and stirred for three hours at RT. Then 335 µL PBS-EDTA-buffer was added and the dispersion stirred for 60 min at RT. Thereafter the dispersion was again magnetically separated as described above but washed and eluted with PBS buffer. As a main fraction yield, 1.55 mL IONPC (*c*(Fe) = 117.8 mmol/L) were obtained.

### 2.14. Synthesis

#### 2.14.1. Chondroitin Sulfate L-Cysteine Conjugate (CS-cys)

The L-cysteine conjugate of CS (CS-cys) was synthesized via a modified 1-Ethyl-3-(3-dimethylaminopropyl)carbodiimide (EDC) mediated coupling reaction between L-cysteine and chondroitin sulfate. Briefly, 1 g CS, MW 50 K (20 µmol) was dissolved in portions in 48.5 mL water at RT and under an argon atmosphere with stirring. Then solutions of 104 mg (0.54 mmol)1-Ethyl-3-(3-dimethylaminopropyl)-carbodiimide-hydrochloride (EDC-HCl) in 0.5 mL water and 31 mg N-hydroxysuccinimide (NHS) (0.27 mmol) in 0.5 mL water were added successively under stirring. The mixture was stirred for 30 min at RT and the pH was adjusted to a value of 5 and kept there using 1 N hydrochloric acid and 1.5 N sodium hydroxide. Then a solution of 53 mg L-cysteine (0.44 mmol) in 0.5 mL water was added and the mixture stirred for 270 min at RT. Thereafter 0.74 g (4.8 mmol) 1,4-dithiothreitol (DTT) was added and the solution stirred for 24 h at RT. The sample was dialyzed using a dialysis tube with a cutoff of 12–14 kDa against 1 L water, mixed with 2 mL of 0.05 N EDTA disodium salt solution for 2 h and then against 1 L water for 18 h (two water changes) and subsequently freeze-dried. The obtained total yield of CS-cys was 780 mg.

#### 2.14.2. Hyaluronic Acid L-Cysteine Conjugate (HA-cys)

L-cysteine conjugate of HA (HA-cys) was synthesized via a modified EDC mediated coupling reaction between L-cysteine and hyaluronic acid. Briefly, 200 mg HA, MW 50 K (4 µmol) was dissolved in portions in 47.5 mL water at RT and under an argon atmosphere with stirring. Then solutions of 21 mg (0.11 mmol) EDC-HCl in 0.5 mL water and 6.2 mg NHS (53.9 µmol) in 0.5 mL water were added successively under stirring. The mixture was stirred for 30 min at RT and the pH was adjusted to a value of 5 and kept there using 1 N hydrochloric acid and 1.5 N sodium hydroxide. Then a solution of 13.2 mg L-cysteine (0.11 mmol) in 0.5 mL water was added and the mixture stirred for 270 min at RT. Thereafter 0.74 g (4.8 mmol) DTT was added and the solution stirred for 24 h at RT. The sample was dialyzed using a dialysis tube with a cutoff of 12–14 kDa against 1 L water, mixed with 2 mL of 0.05 N EDTA disodium salt solution for 2 h and then against 1 L water for 18 h (two water changes) and subsequently freeze-dried. The obtained total yield of HA-cys was 180 mg.

#### 2.14.3. Synthesis of HA-Specific Iron Oxide Nanoparticles (HAIONPs)

For the synthesis of HAIONPs, 2.5 mL of synomag^®^-D (*c*(Fe) = 86.6 ± 0.35 mmol/L) were mixed with 275 µL of 10× PBS-EDTA buffer (100 mM sodium Phosphates, 10 mM Na_2_EDTA) and 9.2 mg Sulfo-SMCC (21.1 µmol) in 170 µL DMSO and stirred for 60 min at RT. Thereafter the dispersion was filled into an LS Column which is placed in a MidiMACS^®^ Separator and pre-washed twice with 5 mL PBS-EDTA-buffer 1×. Then the IONP-loaded LS Column was washed with 5 mL PBS-EDTA-buffer 1×, removed from the MidiMACS^®^ Separator and the activated synomag^®^-D were eluted with 5 mL PBS-EDTA-buffer 1×. A 2.60 mL main fraction was obtained. Pre-run and post-run were each discarded. The main fraction was then mixed with a solution of 2.82 mg HASP (Mw = 1788.49 g CGGKRDLSRRA, TFA salt) in 1.5 mL PBS-EDTA-buffer and stirred for three hours at RT. Then 335 µL of a 20 mM L-cysteine solution in PBS-EDTA-buffer was added and the dispersion stirred for 60 min at RT. Thereafter the dispersion was again magnetically separated like described above, but washed and eluted with PBS buffer. As a main fraction yield, 1.52 mL HAIONP (*c*(Fe) = 112.1 ± 2.2 mM) with a peptide content of 0.435 ± 0.035 mg/mL were obtained.

## 3. Results and Discussion

### 3.1. Synthesis of HAIONPs

**Figure 1:**
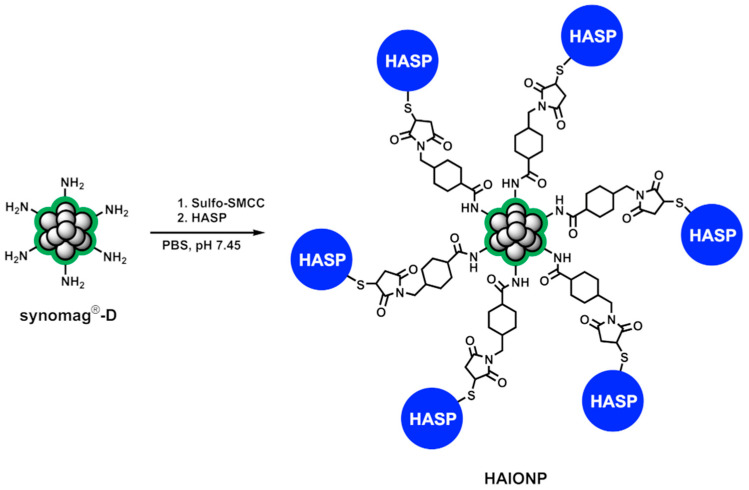
Synthesis of HA-specific iron oxide nanoparticles (HAIONPs) via conversion of synomag^®^-D with Sulfo-SMCC crosslinkers and following addition of HA-specific peptides (HASP).

HASP is based on a peptide called IP3 which was originally identified by in vivo high-throughput sequencing (HTS) using phage display in a mouse model of peritoneal carcinosis of gastric origin and contains an HA-binding motif [14,15,17]. According to the literature, IP3 targets the extracellular matrix of tumors and macrophages with simultaneous very low affinity to the kidneys [14]. For our purposes, starting from IP3, we exchanged one cysteine at the C-terminus with an alanine and added two glycine as a spacer between the cysteine and the lysine at the N-terminus to obtain HASP. Synomag^®^-D [35] were reacted with Sulfo-SMCC in PBS-EDTA buffer and thereafter the activated IONPs were converted with HASP (Figure 1) [36].

Potentially unreacted maleimides of the Sulfo-SMCC linker were converted with excess cysteine to prevent their reaction with free amino groups of the peptide, which would result in a reduction in affinity towards hyaluronic acid. After each synthesis step, the mixture was purified with the aid of LS Columns in a MidiMACS^®^ Separator by binding with the aid of a magnet, followed by washing and then elution after removing of the magnet.

### 3.2. Characterization of HAIONPs

**Figure 2:**
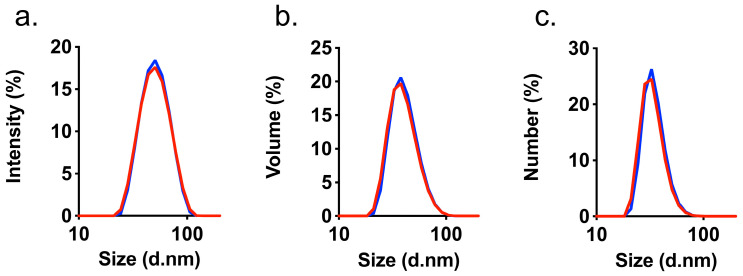
DLS measurements: comparison of synomag^®^-D (red) and HA-specific iron oxide nanoparticles (HAIONPs) (blue) by intensity (**a**), volume (**b**) and numbers (**c**).

After synthesis, the iron content of the resulting dispersion was measured, and the peptide content was determined to prove the successful coupling of HASP. The latter measurement which was carried out using a BCA assay [37,38] indicated a concentration of 0.435 ± 0.035 mg HASP per mL of HAIONP dispersion which corresponds to 0.07 mg peptide per mg of iron or 23% of the used HASP during synthesis (for BCA results please see Supplement Figure S1). In the calibration curve for this assay, the synomag^®^-D parent IONPs were used as background and HASP as standard. Dynamic Light scattering (DLS) [39] measurements show no significant differences between synomag^®^-D parent IONPs and HAIONPs (Figure 2) and therefore no increase in hydrodynamic sizes which would indicate aggregate formation during synthesis, which would be serious for the planned subsequent use in biological systems [40]. Resulting HAIONP dispersions in PBS were stable over several months at 4 °C. Since the measured zeta potential is quite low, the observed stability must be mainly caused by steric stabilization [40,41].

This result was to be expected, as the HASP linker construct is too small to cause a significant change in hydrodynamic diameter after synthesis. As summarized in Table 1, from DLS, narrow size distributions followed for both systems that differ in (volume weighted) hydrodynamic values d_V_ by only 0.9 nm, which is not significant. The *ζ*-potential [39] of HAIONPs became more negative after conversion. Also determined were the relaxivities *r*_1_ and *r*_2_, measured by TD-^1^H NMR. The high *r*_2_ values in the range *r*_2_ = 420–430 mmol^−1^s^−1^ make the system suitable for *T*_2_- or *T*_2_*-weighted MRI applications [42,43].

**Figure 3:**
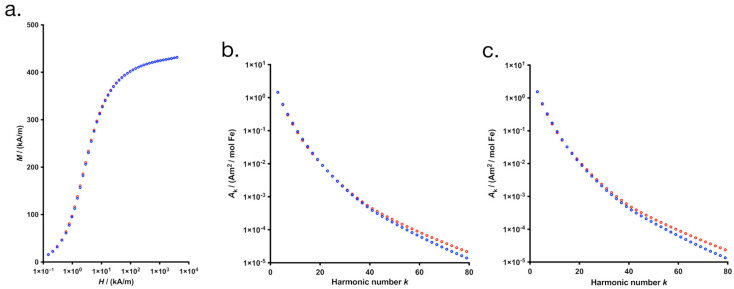
Magnetization of synomag^®^-D (red) and HA-specific iron oxide nanoparticles (HAIONPs) (blue) depending on the applied magnetic field and normalized to the volume fraction *ϕ* of the core material (**a**). Magnetic particle spectroscopy (MPS) data of synomag^®^-D (red) and HA-specific iron oxide nanoparticles (HAIONPs) (blue), measured at *B* = 25 mT and *f*_0_ = 25 kHz (**b**). Magnetic particle spectroscopy (MPS) data of synomag^®^-D (red) and iron oxide nanoparticles control sample (IONPC) (blue), measured at *B* = 25 mT and *f*_0_ = 25 kHz (**c**).

The further magnetic characterization was conducted by DCM and MPS measurements. The magnetization curves nearly show saturation behavior with an approximate saturation magnetization *M*_S_ = 420 kA/m for both systems, indicating that there is no significant difference in *M*s between synomag^®^-D parent IONPs and HAIONPs (Figure 3a). However, at low fields (*H* = 1–10 kA/m) the magnetization of HAIONPs is reduced by about 4%.

In MPS measurements, especially higher harmonics *A*_k_ of HAIONPs are found to be below the amplitudes of the synomag^®^-D parent IONP system (Figure 3b). MPS measures the nonlinear magnetic response of a sample to a homogeneous, alternating magnetic field, thus it is regarded as zero-dimensional MPI, providing a measure of the signal strength (from absolute amplitudes) and resolution (from the degree of decrement of the spectrum *A*(*k*)) of MPI-images for the given IONPs [44]. Based on the MPS spectrum, it can be assumed that HAIONPs are very well suited for MPI, because the amplitude of its *A*_3_ is three times as high as that of Resovist^®^ [45], but of course this still needs to be verified in an MPI scanner in the three-dimensional case. The MPS amplitudes of HAIONPs are slightly smaller than those of the synomag^®^-D parent IONPs, especially for higher harmonics (Figure 3b). Obviously, this is related to the reduced quasistatic magnetization at low fields. Furthermore, the NMR-relaxivities *r*_1_ and *r*_2_ of HAIONPs are reduced (Table 1). This seems to be related to its slightly lower magnetic susceptibility, too. This reduction in magnetic response probably originates from the twofold treatment by magnetic separation columns during synthesis, because the IONPs belonging to the large size tail of the size distribution were retained more effectively because of its larger magnetic moment. It might be that they even got bound to the column irreversibly so that they are absent in HAIONPs. To prove this, we carried out a control experiment and processed synomag^®^-D in two separation steps with the magnetic columns, but without the actual synthesis. And indeed, we observed a similar decrease in the higher harmonics of PCS (Figure 3c), which is a strong indication that the processing using the magnetic columns in the cleaning step changes the size distribution of the magnetic cores, slightly, and thus the magnetic behavior as suspected.

### 3.3. QCM Affinity Measurements

**Figure 4:**
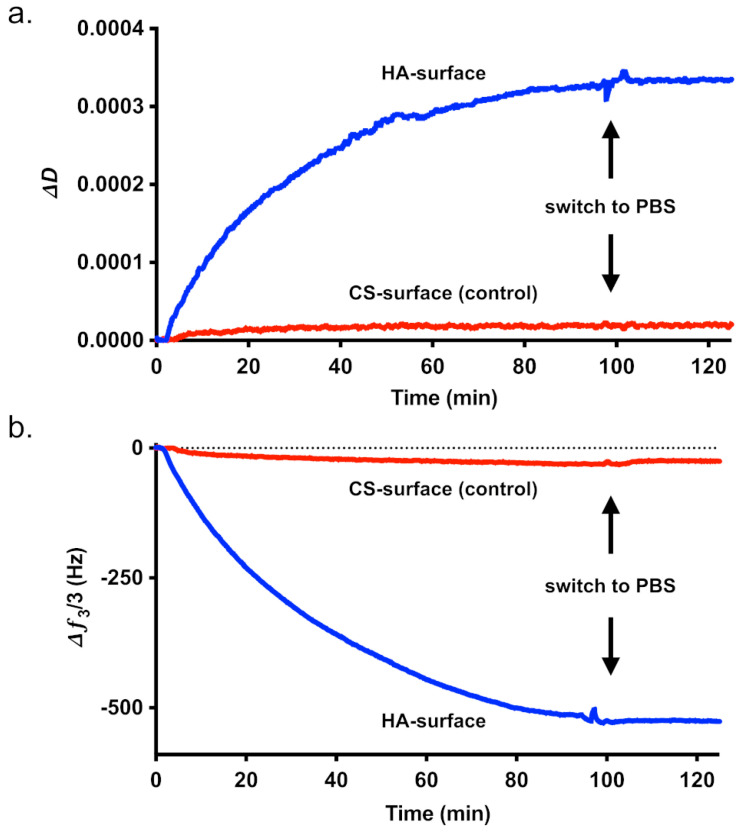
Quartz crystal microbalance (QCM) measurements of dissipation (**a**) and frequency (**b**) shifts in HA-specific iron oxide nanoparticle (HAIONP) deposition on HA- and CS-coated 10 MHz QCM sensors; 3rd harmonics normalized (∆*ƒ*_3_/3).

To test the affinity of HAIONPs to HA, we used a QCM with gold-coated 10 MHz sensors. For this purpose, the carboxylate groups of the two GAGs, HA and CS were activated with EDC and NHS and reacted with cysteine to serve as a coating material for the gold sensor surface [46,47]. The products HA-cys and CS-cys were analyzed by FTIR and compared with the starting materials. Since only a small amount of cysteine was added, no typical thiol bands in the range 2550–2600 cm ^−1^ were visible (for FTIR spectra please see Supplement Figures S2 and S3). To prove the reaction with the cysteine, an Ellmann assay was used to detect the free thiol groups. A degree of substitution (DS) in the range between 7.1 ± 0.004 and 15.6 ± 0.026 was obtained for HA-cys and CS-cys which is sufficient for a strong binding to the gold sensor surface. After coating, the binding affinity of the HAIONP to the respective GAG-coated sensor was measured under flow conditions. Figure 4 shows examples of affinity measurements of HAIONPs using QCM sensors coated with HA-cys or CS-cys, respectively.

Displayed are the frequency and dissipation change over time. In contrast to the control measurement with CS, the QCM results show a binding of the HAIONPs to the sensors coated with HA. After adding equal amounts of HA-cys and CS-cys, we measured a frequency decrease of about Δ*f*_3_ = −518 Hz in the case of HA and only about Δ*f*_3_ = −30 Hz in the case of CS control, which is a clear indication for a strong binding of HAIONPs to HA. The non-specific interaction between HAIONPs and CS on the sensor surface is only 6% of the interaction with HA. After subsequent washing with PBS, the frequency shift is reduced by 18% in the case of CS, whereas no significant change occurs in the case of HA. Table 2 shows the averaged results of the QCM measurements performed (please see also Supplement Figure S4).

### 3.4. MPS Measurements of HAIONP Adsorption on GAG-Coated QCM Sensors

#### 3.4.1. MPS Measurement of the Adsorption of HAIONPs on GAG-Coated QCM Sensors Without Flow

We measured the amount of HAIONPs which adsorbed within 60 min on the gold surfaces of QCM sensors coated with HA-cys and CS-cys, respectively, by MPS measurement of the supernatants (please see Supplement Figure S5). In the case of the HA-coated surface no HAIONPs (less than 1 ng detection limit) could be detected by MPS in the supernatant, so nearly 100% of HAIONPs were bound to the surface, and no HAIONPs were detected in the HA-wash sample, as well. In the supernatant of the CS-coated surface there were about 20% of the HAIONP amount of the stock dispersion detected and again no HAIONPs could be detected in the CS-wash sample. We observed no change in the MPS signal shape parameter *A*_5_/*A*_3_ indicating that the dynamic properties of the IONPs were not changed (due to aggregation) after contact with the HA or CS surface.

The QCM experiments also revealed that there is a small affinity of HAIONPs to CS-coated surfaces; therefore, if the amount of HAIONPs provided is small, the adsorption of HAIONPs can be measured by extremely sensitive methods like MPS. One challenge of the adsorption measurement method used is that the HAIONPs may behave differently in the static case without flow than in the QCM measurement, and that the coated surfaces are very sensitive to contact, which can hardly be avoided, e.g., when removing the supernatant by pipette and may therefore affect the measurement result.

#### 3.4.2. MPS Measurement of the Adsorption of HAIONPs on GAG-Coated QCM Sensors After QCM Affinity Measurements

**Figure 5:**
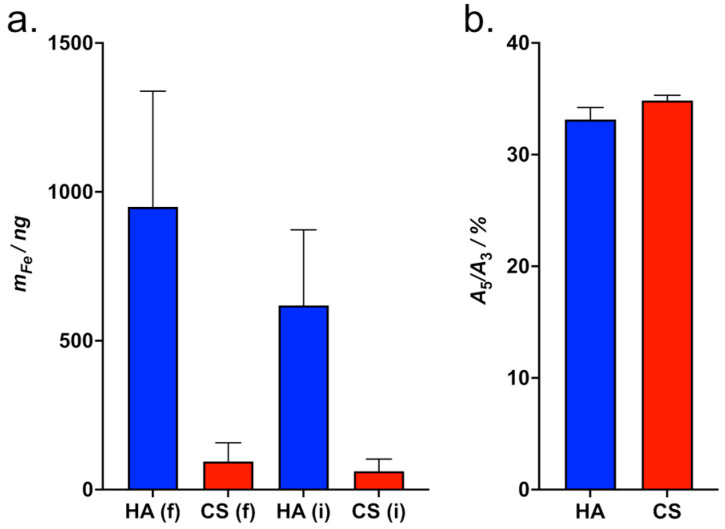
MPS measurements of the adsorption of HA-specific iron oxide nanoparticles (HAIONPs) on the HA- and CS-coated QCM sensors after affinity measurements. HAIONPs were removed with cellulose tissue and measured using MPS. Given are the MPS results of iron mass *m* evaluated with a fluid HAIONP reference: HA (f) and CS (f) and with an immobilized HAIONP reference: HA (i) and CS (i) (**a**), as well as the ratios of MPS signal amplitudes *A*_5_/*A*_3_ of HA and CS (**b**), measured at *B* = 25 mT and *f*_0_ = 25 kHz.

To confirm the amounts of HAIONPs adsorbed onto the GAG-coated QCM sensors during the affinity measurements, we removed the HAIONPs from the QCM sensor surfaces using cellulose tissue after the experiments and measured them via MPS (Figure 5).

With a measured mean amount of ten times more iron on the HA-coated sensors than on the CS-coated sensors in both cases, evaluated with a fluid and an immobilized reference, this method also demonstrated the much higher affinity of HAIONPs to HA compared to CS. Whereby it is unclear whether the removal of HAIONPs from the sensor surface was quantitative and whether, during the affinity measurements, all HAIONPs were actually removed from the measuring chamber inside of the QCM by rinsing with PBS. The latter in particular could distort the results. The *A*_5_/*A*_3_ ratios (Figure 5b), which are a measure of the state of HAIONPs, differ slightly from each other and significantly from the stock solution (please see Supplement Figure S5). These differences can be explained by the interactions of HAIONPs with HA and CS on the one hand and the cellulose tissue on the other.

**Figure 6:**
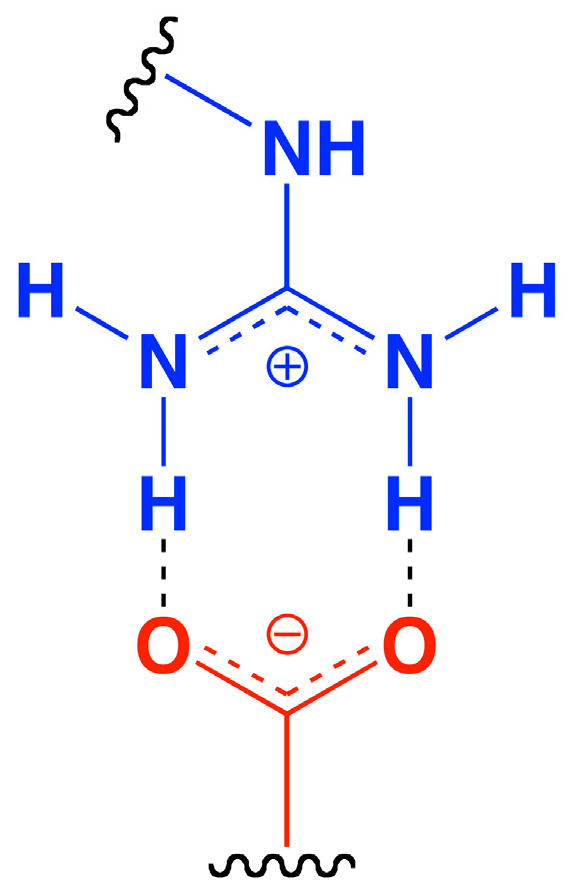
One of the possible salt bridges between a carboxylic group of HA (red) and a guanidine group of arginine (blue).

The literature describes that the binding affinity of peptides to HA depends on certain patterns in the sequence of basic and neutral or acidic amino acids [14,15,17]. The distance between the basic amino acids seems to be decisive for the binding affinity to HA and is probably complementary to the distance of the carboxylic groups of HA in the preferred conformation. Carboxylic groups can form so-called salt bridges, particularly between the carboxyl groups of HA and the guanidine groups of arginine, which in combination with other interactions such as further hydrogen bonds, could probably lead to a high overall affinity for HA (Figure 6) [48,49].

A single peptide would only cause weak binding due to its limited number of interactions to HA, and then the unspecific interactions of the IONP surface would dominate. But since many peptides are bound to the IONPs, the IONPs can bind multivalently to HA, resulting in a strong binding and thus increasing the affinity and selectivity [50]. However, the question arises as to the optimal HASP loading density of HAIONPs. If the loading density is too low, the affinity for HA and therefore selectivity could be low. However, if the loading density is too high, it may be that no lateral interaction of the peptides with HA can occur, thus significantly reducing the binding affinity. The spacer length can also play a role in terms of affinity, as a peptide that is further away from the IONP surface has theoretically more degrees of freedom for optimal orientation for interaction with HA. After coating with GAGs, the potential unoccupied binding sites of the gold sensor surface were blocked with L-cysteine to avoid unspecific binding. For evaluation of the binding of HAIONPs to the respective GAGs by QCM, not the fundamental frequency but the third harmonic was used, as the higher harmonics do not reach as far into the adjacent medium as the fundamental frequency due to the higher frequency of the sensor surface, and therefore better reflect the changes near the sensor surface and are therefore less susceptible to interference [51].

## 4. Conclusions and Outlook

The attachment of HASP to the amine groups of synomag^®^-D via the SMCC linker was successfully achieved. The QCM binding experiments carried out showed that HAIONPs have a very high affinity for HA-coated QCM sensors in contrast to CS-coated ones. The MPS measurements of the HAIONP quantities present on the sensors after QCM affinity measurements also showed a tenfold higher iron value on the HA coated QCM sensors compared to the CS coated ones. These results suggest that the number of peptides incorporated within HAIONPs are sufficient to enable high affinity through multivalent binding, although the occupancy of the HAIONP surface with peptides is apparently still so low that they can bind laterally to HA. Furthermore, it can be stated that it is important to use IONPs with a narrow magnetic size distribution in syntheses if magnetic columns are to be used for purification, so that the particles are not altered too much by this procedure. Synthesized HAIONPs are stable in PBS over longer periods of time without aggregate formation and therefore potentially suitable for biomedical applications. As a next step, investigating the HAIONP binding to HA-rich cell surfaces in MPI is planned to see whether the promising binding results of HAIONPs to HA obtained in the QCM experiments can be confirmed in vitro. Furthermore, it can also be assumed that HAIONPs should not only be suitable as an MPI tracer [35] or MRI contrast agent but also well suited for magnetic fluid hyperthermia applications [52,53]. The latter can also be combined with MPI for theranostic applications using corresponding devices [52,54]. The magnetic properties combined with the high affinity for HA make HAIONPs potentially a promising probe for imaging and investigating of pathologic extracellular matrix changes. The combination of QCM with MPS can become a powerful tool for the development of magnetic probes for the sensitive detection of changes in ECM components.

## Figures and Tables

**Table 1:** Properties of HA-specific iron oxide nanoparticles (HAIONPs) and synomag^®^-D. Shown are the relaxivities *r*_1_ and *r*_2_ and the corresponding values from the DLS measurement, the mean hydrodynamic diameter by volume (d_V_), the intensity-weighted mean hydrodynamic size (Z-Average), the polydispersity index (PDI) and the zeta potential (ζ).

IONPs	*r*_1_ L mmol^−1^s^−1^	*r*_2_ L mmol^−1^s^−1^	d_V_ DLS nm	Z-Average nm	PDI	ζ-Potential mV
synomag^®^-D	14	432	41.0 ± 12.83	48.0	0.082	−1.77 ± 6.07
HAIONPs	13	421	41.9 ± 12.59	48.5	0.071	−2.82 ± 9.02

**Table 2:** Results of QCM affinity measurements. Frequency shifts in Hz after addition of HAIONPs and subsequent addition of PBS.

	1. HAIONP Addition		2. PBS Addition	
GAG	HA	CS	HA	CS
∆*ƒ*_3_/3 (Hz)	−548 ± 33	−21 ± 7	11 ± 13	7 ± 2

## Data Availability

The data presented in this study are available on request from the corresponding author.

## Supplementary Materials

The following supporting information can be downloaded at https://www.mdpi.com/article/10.3390/nano15191505/s1, Figure S1: BCA Measurement; Figure S2: FTIR spectra of HA and HA-cys; Figure S3: FTIR spectra of CS and CS-cys; Figure S4: QCM frequency shifts upon addition of 1. HAIONPs and 2. PBS; Figure S5: MPS measurements of the adsorption of HA-specific iron oxide nanoparticles (HAIONPs) on the HA- and CS-coated gold surfaces, static case without flow.

## Abbreviations

The following abbreviations are used in this manuscript:

BCA	bicinchoninic acid
CS	chondroitin sulfate
CS-cys	chondroitin sulfate L-cysteine conjugate
DCM	direct current magnetization
DLS	dynamic light scattering
DTT	1,4-dithiothreitol
ECM	extracellular matrix
EDC	1-ethyl-3-(3-dimetylaminopropyl)-carbodiimide
EDTA	ethylenediaminetetraacetic acid
GAGs	glycosaminoglycans
HA	hyaluronic acid
HA-cys	hyaluronic acid L-cysteine conjugate
HAIONP	HA-specific iron oxide nanoparticle
HAS	HA synthases
HASP	HA-specific peptide
IONP	iron oxide nanoparticle
IONPC	IONP control sample (treated with magnetic columns)
MPI	magnetic particle imaging
MPS	magnetic particle spectroscopy
MRI	magnetic resonance imaging
n.d.	not determined
NHS	N-hydroxysuccinimide
NMR	nuclear magnetic resonance
PBS	phosphate-buffered saline
QCM	quartz crystal microbalance
RT	room temperature
Sulfo-SMCC	sulfosuccinimidyl-*trans*-4-(N-maleimidomethyl)cyclohexane-1-carboxylate
