# Supplementary material for: Synthesis and Characterization of a Nanoscale Hyaluronic Acid-Specific Probe for Magnetic Particle Imaging and Magnetic Resonance Imaging
Source: Nanomaterials (Basel). 2025 Oct 1;15(19):1505. doi: 10.3390/nano15191505 (PMC12526343; doi:10.3390/nano15191505)
Supplement: Supplementary file 1 [file nanomaterials-15-01505-s001.zip › Figure S5.pdf]

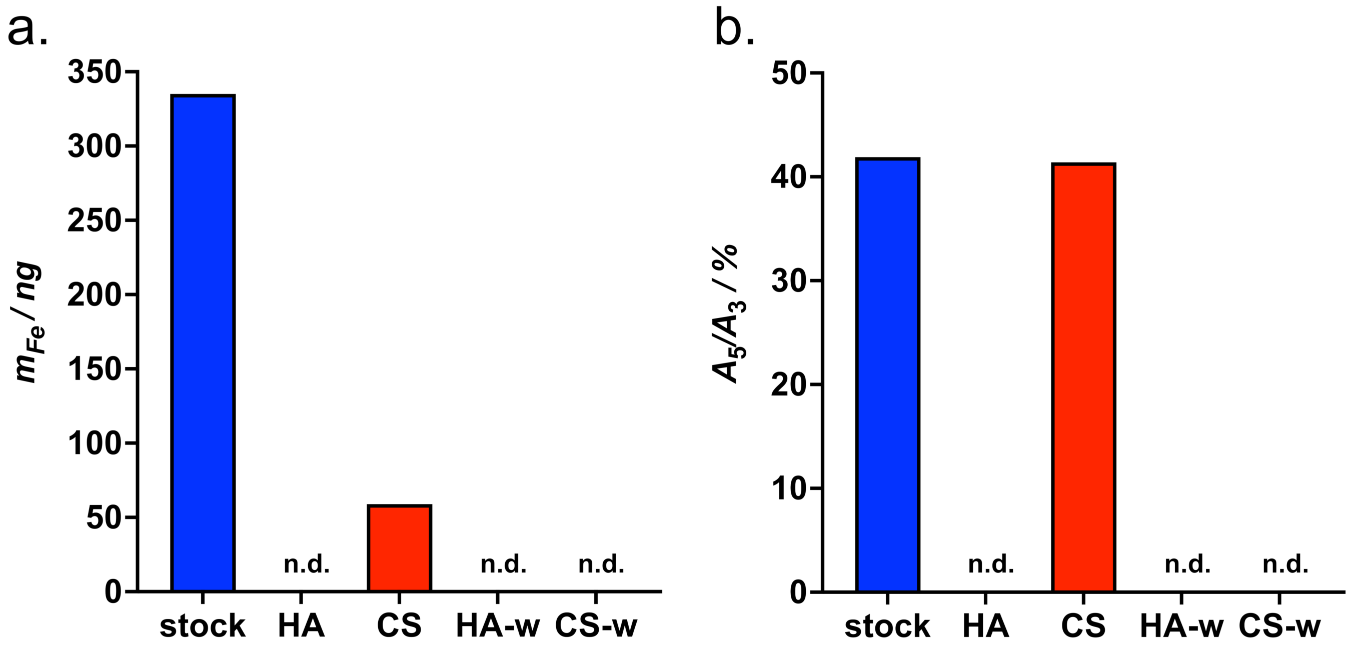

Figure S5. MPS measurements of the adsorption of HA-specific iron oxide nanoparticles (HAIONP) on the HA- and CS-coated gold surfaces. Given are the MPS results of iron mass m (a.) and ratio of MPS signal amplitudes A5/A3 (b.), of the five dispersions: HA-specific iron oxide nanoparticles (HAIONP) stock (stock), HA, CS, HA-wash (HA-w), and CS-wash (CS-w), measured at B=25 mT and f0=25 kHz, n.d. means not determined.
