# Supplementary figures and images for: Synthesis and Characterization of a Nanoscale Hyaluronic Acid-Specific Probe for Magnetic Particle Imaging and Magnetic Resonance Imaging
Source: Nanomaterials (Basel). 2025 Oct 1;15(19):1505. doi: 10.3390/nano15191505 (PMC12526343; doi:10.3390/nano15191505)

**Figure S1. BCA Measurement**

**HASP: BCA calibration curve**

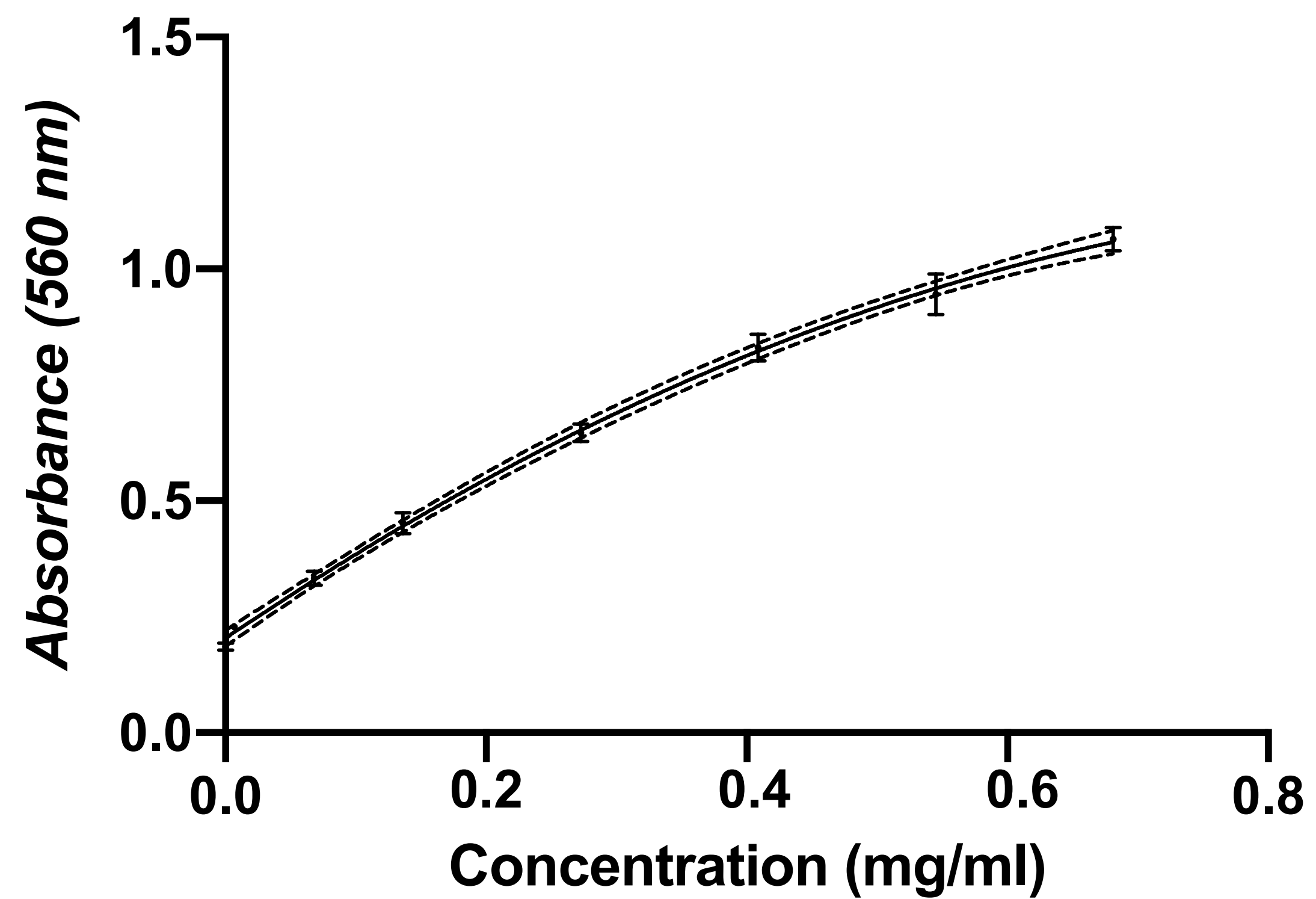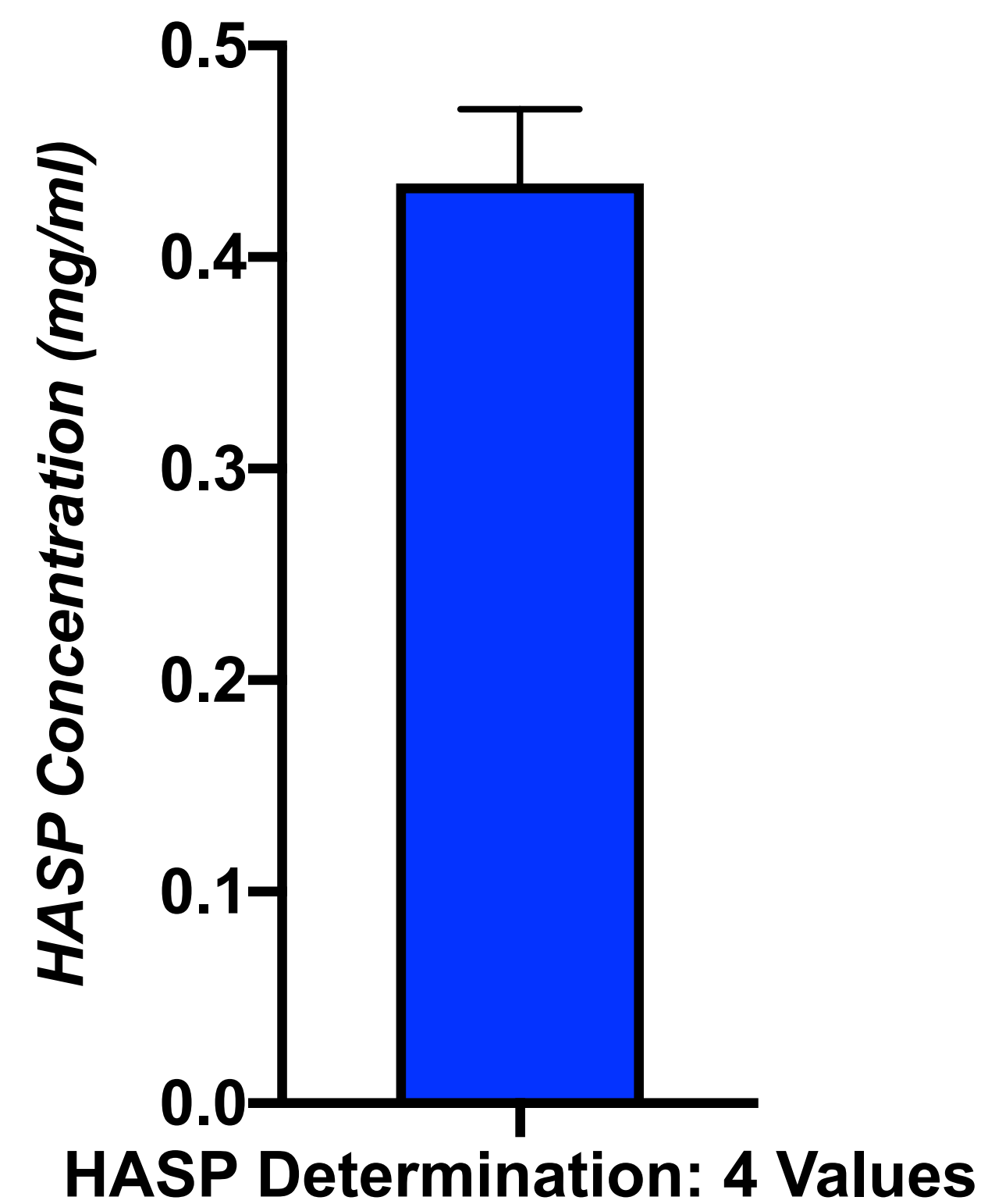

Supplement: Supplementary file 1 [file nanomaterials-15-01505-s001.zip › Figure S1.pdf]

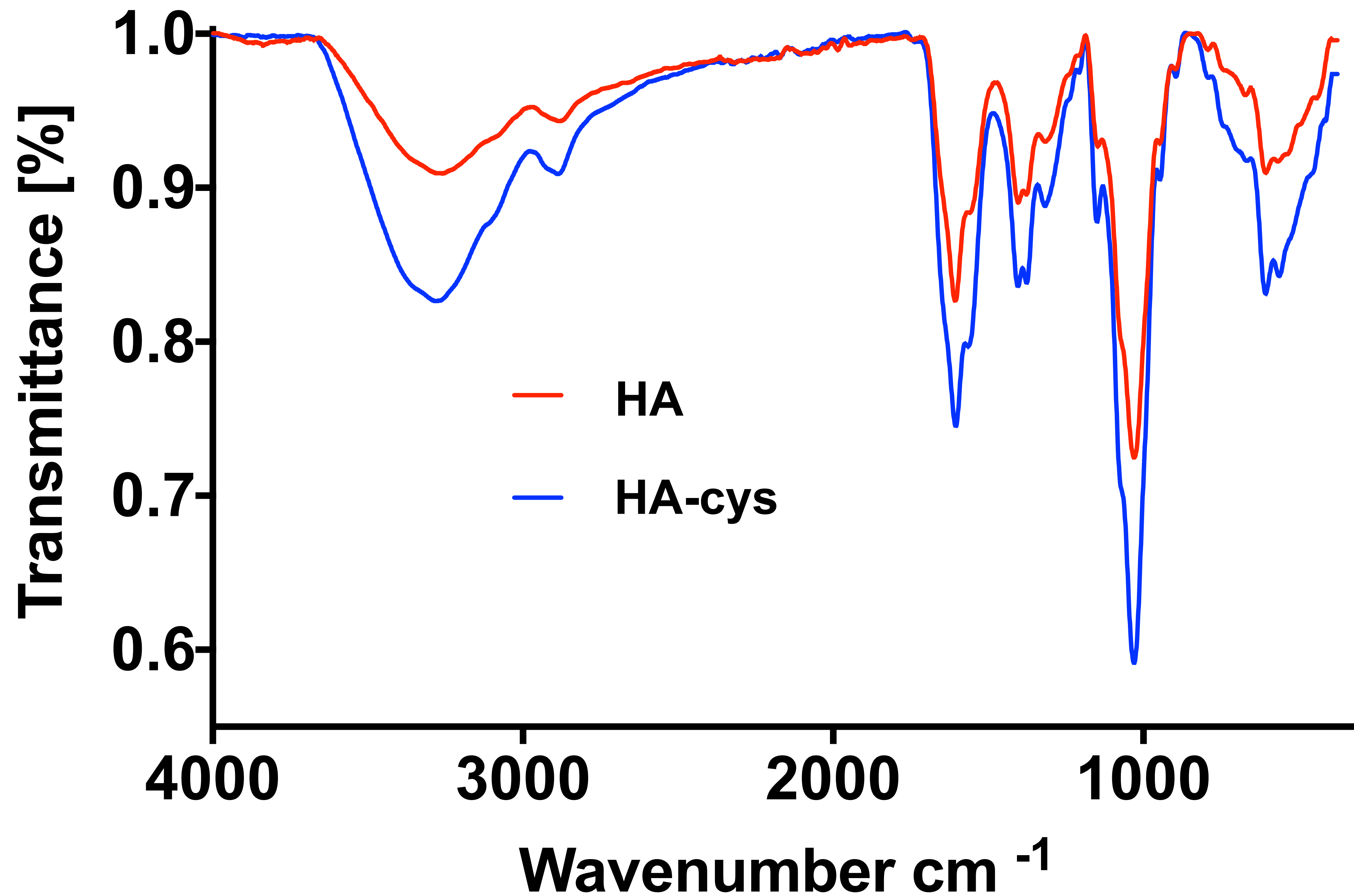

Figure S2. FTIR spectra of HA and HA-cys

Supplement: Supplementary file 1 [file nanomaterials-15-01505-s001.zip › Figure S2.pdf]

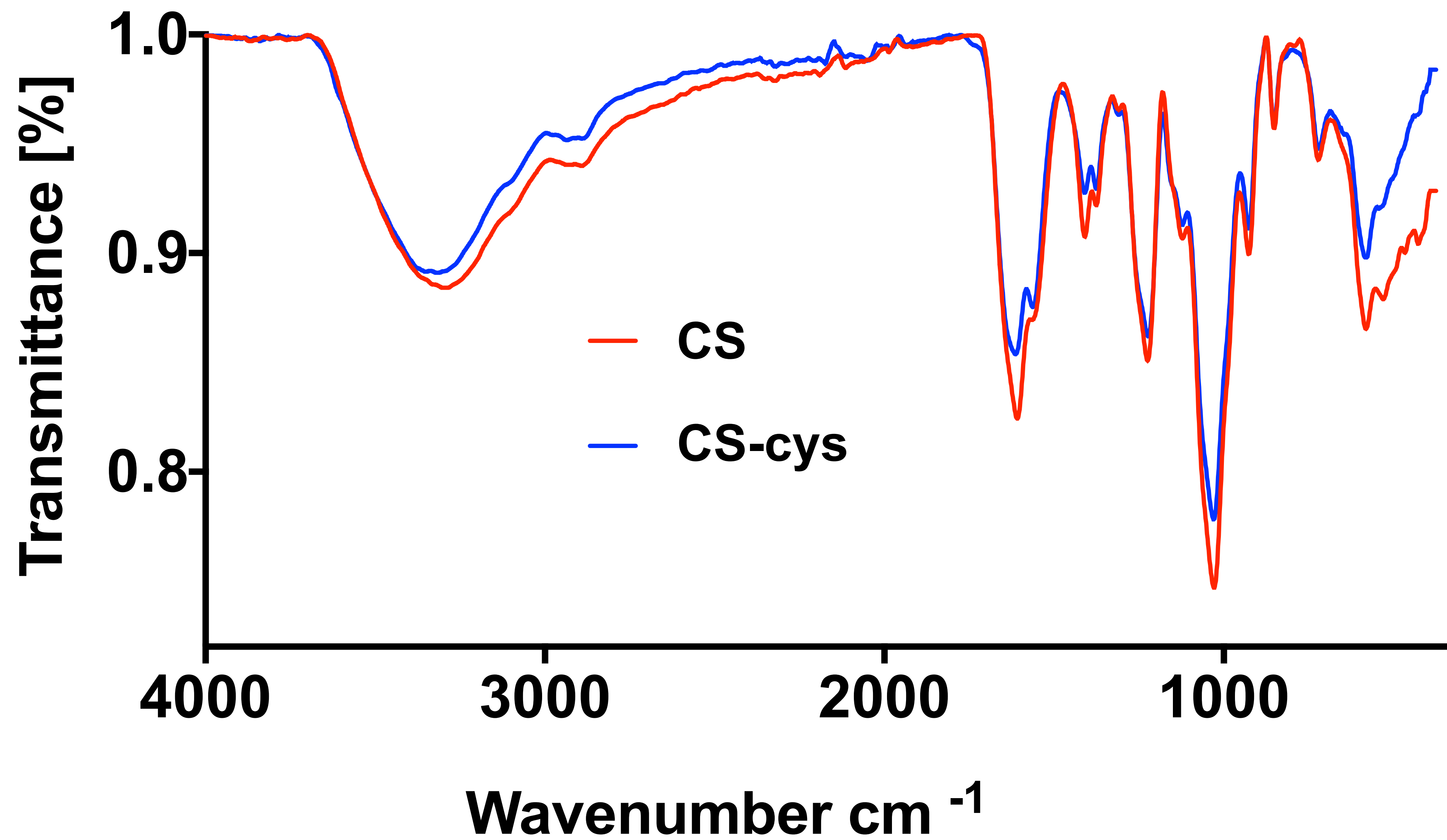

Figure S3. FTIR spectra of CS and CS-cys

Supplement: Supplementary file 1 [file nanomaterials-15-01505-s001.zip › Figure S3.pdf]

Figure S4. QCM frequency shifts upon addition of 1. HAIONP and 2. PBS

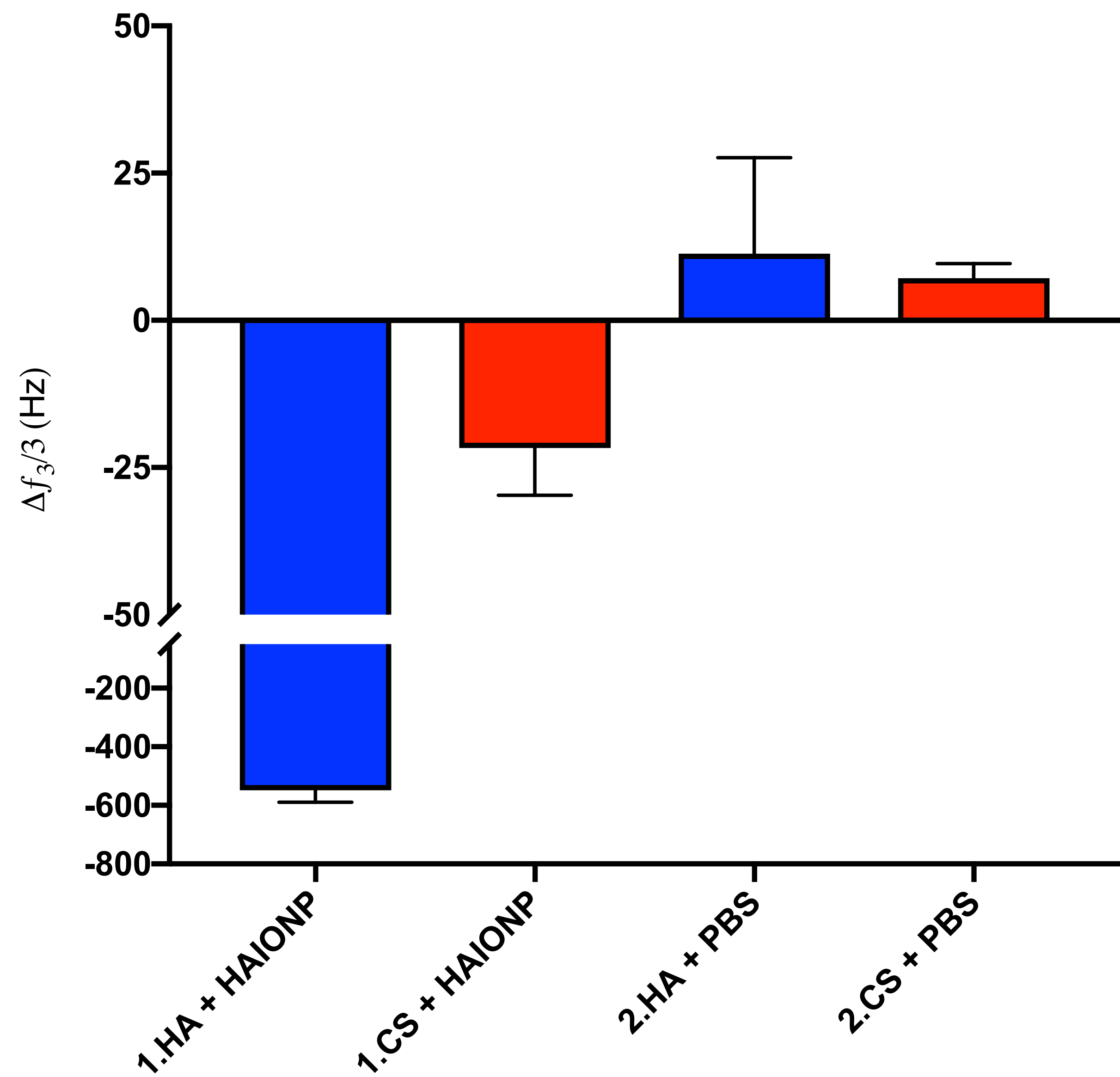

Supplement: Supplementary file 1 [file nanomaterials-15-01505-s001.zip › Figure S4.pdf]
